# Bilateral Carotid Artery Dissection After a Fall: A Case of Horner Syndrome Revealed on Examination

**DOI:** 10.5811/cpcem.47151

**Published:** 2025-08-24

**Authors:** Eli Spevack, Zachary M Weisner, Evgenia Nokovich, Michelle Joyner, Lauren Exley

**Affiliations:** * Philadelphia College of Osteopathic Medicine, Philadelphia, Pennsylvania; † Jefferson Northeast, Graduate Medical Education EM/IM Residency, Department of Internal Medicine, Department of Emergency Medicine, Philadelphia, Pennsylvania; ‡ Jefferson Northeast, Department of Emergency Medicine, Philadelphia, Pennsylvania

**Keywords:** case report, trauma, carotid dissection, Horner syndrome

## Abstract

**Introduction:**

Carotid artery dissections are uncommon but critical vascular injuries. They involve a tear to the intima, the innermost layer of the arterial wall, leading to formation of a false lumen. This false lumen can disrupt blood flow, weaken the wall, and lead to thrombus or rupture of the artery. Carotid artery dissections can occur spontaneously or in the setting of trauma. Traumatic carotid artery dissections (TCAD) are rare and occasionally present with third-order Horner syndrome, characterized by ipsilateral ptosis, miosis, and anhidrosis. The presence of subtle physical exam signs like Horner syndrome reinforces the importance of maintaining a high index of suspicion and obtaining vascular imaging in trauma-related cases. While there have been case reports of bilateral TCAD, these have been rarely reported in the literature.

**Case Report:**

We present a case involving a 53-year-old female with no significant past medical history who presented to the emergency department after tripping and falling down a flight of stairs. Over three weeks, the patient had persistent tinnitus and right neck pain and, on the exam, was found to have right-sided miosis and ptosis. These exam findings led us to obtain a computed tomography (CT) angiogram of her neck, which revealed bilateral internal carotid artery dissections. The patient was taken for cerebral angiography, which confirmed the diagnosis. A stent was placed in the right internal carotid artery, and the patient was started on aspirin and clopidogrel. The patient was discharged without deficits three days later.

**Conclusion:**

Traumatic internal carotid artery dissection can occasionally result in Horner syndrome and requires CT angiography of the neck and potentially a diagnostic cerebral angiogram to diagnose. This case adds to the limited literature on bilateral TCAD, particularly with a delayed and asymmetric presentation. Horner syndrome in the setting of trauma, while subtle, can suggest a carotid artery dissection. Awareness of such rare presentations is key to early diagnosis and treatment. Clinicians must maintain a high index of suspicion for underlying vascular injury in patients presenting with lesser mechanisms of injury.

## INTRODUCTION

Carotid artery dissections are uncommon but important vascular injuries. They involve a tear to the intima, the innermost layer of the arterial wall, leading to formation of a false lumen. This false lumen can disrupt blood flow, weaken the wall, and lead to thrombus or rupture of the artery. Carotid artery dissections can occur spontaneously or in the setting of trauma. Traumatic carotid artery dissections (TCAD) are rare and occasionally present with third-order Horner syndrome, characterized by ipsilateral ptosis, miosis, and anhidrosis. The presence of subtle physical exam signs like Horner syndrome reinforces the importance of maintaining a high index of suspicion and obtaining vascular imaging in trauma-related cases. The annual incidence of CADs occurs at a rate of 2.6 per 100,000 people.^1^,^2^ Horner syndrome occurs in only 25% of these CAD cases. Among all CADs, internal carotid artery dissections (ICAD) are most associated with Horner syndrome. While there have been case reports of bilateral TCAD, rarely have they been reported in the literature.

## CASE REPORT

We present a 53-year-old Spanish-speaking female with no significant past medical history who presented to the emergency department with right-sided neck pain after a fall. The patient reported falling down seven to eight stairs approximately three weeks earlier. Since that fall, she noticed she had issues with her right eye and had also been experiencing left-sided tinnitus. In addition, the patient had sustained a contusion to her left ankle but was able to ambulate without difficulty.

On physical exam, right-sided ptosis and miosis were evident. The pupils were unequal and reactive to light bilaterally. Visual acuity was intact, and extraocular eye motion testing was normal. A whisper test did not reveal any hearing deficits. Cranial nerve examination included assessment of facial sensation, motor function of the cheeks and jaw, and facial symmetry with smile, frown, eyebrow raise and tight eye closure. Gag reflex, swallowing ability, shoulder shrug, and tongue movement were also evaluated. All findings were within normal limits. Neck tenderness was noted from the third and fourth cervical midline and bilateral paraspinal areas.

**Image 1:**
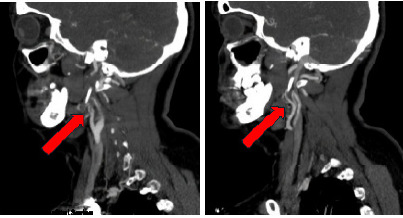
Computed tomography angiography of the neck in sagittal view showing bilateral internal carotid artery dissection (red arrows).

**Image 2:**
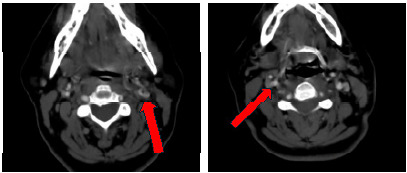
Computed tomography angiography of the neck in transverse view showing bilateral internal carotid artery dissection (red arrows).

With the presence of ptosis and miosis following trauma, there was concern for Horner syndrome. Due to the mechanism of injury and neurologic findings, a computed tomography (CT) angiography of the neck was obtained. Imaging revealed severe stenosis at the distal right internal carotid artery near the skull base, as well as severe stenosis of the mid- and distal left internal carotid artery, all concerning for dissection (Images 1 and 2).

**Image 3:**
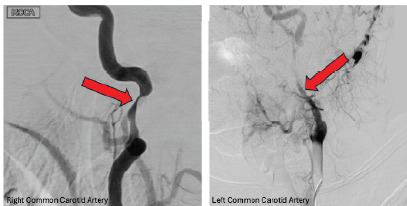
Cerebral angiogram confirming bilateral internal carotid artery dissection (red arrows), with the right being worse than the left.

Neurosurgery was consulted, and the patient was loaded with aspirin and clopidogrel before being transferred to our central hospital for further evaluation. Cerebral angiography confirmed bilateral dissections of the internal carotid arteries (Image 3). A stent was placed in the petrous segment of the right carotid artery, and the patient was admitted to the intensive care unit with a goal mean arterial pressure of < 100 millimeters of mercury. She was maintained on aspirin and clopidogrel. The patient was discharged home after three days of hospitalization without any neurological deficits.

## DISCUSSION

This case contributes a valuable addition to our understanding of bilateral TCAD. In one case of bilateral TCAD that occurred following a devastating motor vehicle crash, the patient presented with anisocoria after being evaluated in the intensive care unit.^3^ Other cases involve similarly high-caliber trauma mechanisms with varying lengths of time to identification of the CAD. Our patient faced a less severe mechanism of injury, and she did not present until three weeks later. In cases with more mild traumatic mechanisms, unlikely conditions such as TCADs may be overlooked but still need to be considered.^4^–^8^

*CPC-EM Capsule*

What do we already know about this clinical entity?

*Carotid artery dissections may follow trauma and present with subtle findings like Horner syndrome or vague head and neck pain*.

What makes this presentation of disease reportable?

*This is a rare case of bilateral carotid artery dissection with a delayed, unilateral presentation of Horner syndrome after a minor trauma*.

What is the major learning point?

*Horner syndrome after trauma can indicate underlying carotid artery dissection. Maintain high suspicion for vascular injury, even with seemingly minor mechanisms*.

How might this improve emergency medicine practice?

*Raise awareness to consider carotid artery dissection in the setting of trauma and Horner syndrome, and obtain computed tomography angiography in patients with those signs*.

Horner syndrome is a condition that can result from a lesion occurring anywhere along the sympathetic pathway supplying the head, neck, and eyes.^9^ The path originates in the hypothalamus. Horner syndrome can be subcategorized as first, second, or third order depending on the location of the lesion.^9^ The first-order neuron descends to the eighth cervical to second thoracic spinal cord levels (C8-T2), where the first synapse occurs.^9^ The second-order neuron travels from the sympathetic trunk, through the brachial plexus, to the superior cervical ganglion, located near the angle of the mandible and carotid artery bifurcation.^9^ The third-order neuron ascends within the internal carotid artery (ICA) adventitia through the cavernous sinus (closely related to cranial nerves VI).^9^ The triad of symptoms associated with Horner syndrome are miosis, ptosis, and anhidrosis.^9^

Coronary artery dissections involve a tear in the intimal layer of the artery, creating an intraluminal hematoma.^10^ In the event of an ICA dissection causing Horner syndrome, typically only miosis and ptosis are present, as facial sweat glands are innervated by sympathetic fibers surrounding the external carotid artery.^10^ With ICADs, the presence of Horner syndrome symptoms is associated with a more favorable course, which includes fewer strokes and better outcomes.^10^

Patients with a traumatic mechanism who complain of any constellation of headache, facial or neck pain, and partial Horner syndrome should be imaged for underlying extracranial CAD. Intra-arterial angiography remains the gold standard for dissection diagnosis as it will demonstrate arterial lumens and vessel wall defects. Magnetic resonance angiographic or T1, T2, and proton density-weighted images are also valuable tools; however, time and accessibility can be a limiting factor. Ultrasound is a noninvasive option, allowing for direct visualization of vessel blood flow as well as true or false lumens.^11^

Treatment of CADs can vary depending on presenting symptoms. If a CAD is detected and the patient has an acute ischemic stroke, intravenous thrombolysis with either alteplase or tenecteplase is appropriate.^12^ In patients with CAD that meet criteria for acute large-vessel occlusion, mechanical thrombectomy should be performed.^12^ In the setting of a CAD causing stenosis or occlusion with hypoperfusion to distal territories, stenting the vessel remains beneficial. Stenting for CADs with the absence of hypoperfusion is controversial and should be considered on an individual basis.^12^ To decrease risk of secondary stroke in the setting of CAD after embolization or stenting, consider antithrombotic therapy.^12^ If there is no elevated bleeding risk and absence of high-risk features (no intraluminal thrombus, non-occlusive dissection), consider dual antiplatelet therapy or monotherapy with aspirin or a P2Y12 inhibitor.^12^ Presence of high-risk features would necessitate parenteral anticoagulation if low bleeding risk followed by oral anticoagulation, or dual/mono antiplatelet therapy for moderate bleeding risk.^12^ Patients should remain on antithrombotic therapy for three to six months.^12^ To avoid recurrent or worsening dissection, patients should avoid minor head/neck trauma in the months following. This includes neck manipulation, extreme sports, heavy lifting, hyperextension, and contact sports.^12^

In this patient, the presence of partial Horner syndrome played a critical role in raising early suspicion of an underlying CAD. This led to prompt imaging, identification, and dual antiplatelet therapy. However, if fewer or no neurological signs had been apparent, there may have been a significant delay in localizing the underlying problem. This patient still complained of neck pain, yet neck or head pain can mimic less severe conditions like musculoskeletal etiologies. An increased index of suspicion must always be present, especially in patients with a recent history of trauma, to avoid misdiagnoses. This risk will be mitigated by conducting a thorough history and a detailed physical exam and having a lower threshold for imaging in trauma patients.

## CONCLUSION

Traumatic internal carotid artery dissection can occasionally result in Horner syndrome and requires CT angiography of the neck and potentially a diagnostic cerebral angiogram to diagnose. This case adds to the limited literature on bilateral TCAD, particularly with a delayed and asymmetric presentation. Horner syndrome in the setting of trauma, while subtle, can suggest a carotid artery dissection. Awareness of such rare presentations is key to early diagnosis and treatment. Physicians must maintain a high index of suspicion for underlying vascular injury in patients presenting with lesser mechanisms of injury.

## References

[1] Lee VH, Brown RD, Mandrekar JN (2006). Incidence and outcome of cervical artery dissection: a population-based study. Neurology.

[2] Schievink WI (2001). Spontaneous dissection of the carotid and vertebral arteries. N Engl J Med.

[3] Cronlein M, Sandmann GH, Beirer M (2015). Traumatic bilateral carotid artery dissection following severe blunt trauma: a case report on the difficulties in diagnosis and therapy of an often overlooked life-threatening injury. Eur J Med Res.

[4] Agarwal A, Yadav D, Gupta A (2020). Delayed bilateral internal carotid artery dissection following motor vehicle accident: time to make its screening a part of trauma protocol?. QJM.

[5] Khormi YH, Darraj AI, Arishy A (2024). Bilateral blunt traumatic dissections of the extracranial internal carotid artery: a case report and literature review. Cureus.

[6] Duncan MA, Dowd N, Rawluk D (2000). Traumatic bilateral internal carotid artery dissection following airbag deployment in a patient with fibromuscular dysplasia. Br J Anaesth.

[7] Busch T, Aleksic I, Sirbu H (2000). Complex traumatic dissection of right vertebral and bilateral carotid arteries: a case report and literature review. Cardiovasc Surg.

[8] Taoussi N, Alghamdi AJ, Bielewicz J (2017). Traumatic bilateral dissection of cervical internal carotid artery in the wake of a car accident: a case report. Neurol Neurochir Pol.

[9] Zhan Z, Bollu PC (2023). Horner Syndrome. StatPearls.

[10] Lyrer PA, Brandt T, Metso TM (2014). Clinical import of Horner syndrome in internal carotid and vertebral artery dissection. Neurology.

[11] Flis CM, Jager HR, Sidhu PS (2007). Carotid and vertebral artery dissections: clinical aspects, imaging features and endovascular treatment. Eur Radiol.

[12] Yaghi S, Engelter S, Del Brutto VJ (2024). Treatment and Outcomes of Cervical Artery Dissection in Adults: A Scientific Statement From the American Heart Association. Stroke.

